# University students’ interest in intervention research participation is associated with gender, age and symptoms of depression, general and social anxiety, panic disorder and self-harm

**DOI:** 10.1186/s13104-026-07785-8

**Published:** 2026-03-29

**Authors:** Elisabeth Tove Irving, Britt Stikvoort, Claes Andersson, Anne H. Berman

**Affiliations:** 1 Department of Psychology, Uppsala University, Uppsala, Sweden; 2 Department of Oncology, Division of Psychosocial Oncology, University of Calgary, Calgary, AB Canada; 3 Department of Criminology, Malmö University, Malmö, Sweden

**Keywords:** Digital interventions, Mental disorder, Treatment gap, Intervention research, University students

## Abstract

**Objective:**

Research in the mental health field has focused on exploration and implementation of digital interventions both in research and society. However, demographic and mental health factors that may influence engagement with research on such interventions have not been explored. This study aims to investigate the relationship between demographics, signs of mental disorders, and interest in participating in an intervention study. Using data collected in the Swedish arm of the World Health Organization’s World Mental Health International College Student (WHO-WMH-ICS) initiative (*n* = 9140), we conducted a multinomial logistic regression to assess relationships between these factors.

**Results:**

Older age, as well as female and non-binary gender identities, were factors significantly associated with increased interest in the intervention study. Treatment flags, indicating fulfilment of criteria for possible mental disorder diagnosis, were significantly associated with clear interest (“yes” response) in intervention research for five of the 15 treatment flags: depression, general anxiety, social anxiety, panic disorder and self-harm. Possible interest (“maybe” response) was also indicated for four of these, excluding general anxiety. These findings may facilitate a better-informed approach to recruiting student participants for treatment research, enhancing unbiased recruitment practices, reducing treatment gaps, and increasing engagement in digital intervention studies for improved mental health.

## Introduction

About half of individuals with common mental disorders do not seek treatment, contributing to the “treatment gap” [1, 2]. Low-threshold digital interventions hold promise to reduce this gap, due to their high accessibility and low stigma [3]. Young adults, half of whom are students in higher education, merit priority in reducing the treatment gap, to promote mental health associated with academic achievement [4] and to enhance navigation of the challenges faced by emerging adults [5]. Research on digital interventions for university students suggest small but promising effects in reducing mental disorders [6].

Notwithstanding identified treatment needs and potentially positive treatment effects of digital interventions, a study of barriers to seeking treatment showed that 75% of the students sampled reported hesitancy in seeking help, due to preferences for resolving mental health problems on their own, or feeling embarrassed [7]. Among these hesitators, those with a current mental disorder had a *higher* likelihood of expressing hesitation than those without, suggesting that the barriers of wishing to solve problems on one’s own and embarrassment overrode the motivation and possible hope of alleviating suffering through possible treatment [7]. Investigation of the relationship between presence of a mental disorder and expressed interest in participating in an actual treatment research study has, to our knowledge, not been reported.

Previous research has identified factors that contribute to an individual’s general willingness to participate in research; for example, confidence in science, education level and age as well as personality traits such as curiosity, self-efficacy and social support [8–11]. Additionally, research has identified gender differences in willingness to participate in research. For instance, women are more likely to participate due to altruistic reasons or the influence of friends, family or researchers [12]. While women tend to be systematically underrepresented in clinical and medical research [13], they constitute most respondents in studies on psychopathology compared to men [14]. We have therefore conducted an exploratory investigation of student interest in a coming intervention research study, with a specific focus on signs of mental disorders and demographic factors, to identify whether students with mental health needs actually show interest in participating in an intervention study, and to gain a deeper understanding of factors that characterize individuals interested in engaging in such research. The findings may facilitate a more informed approach to recruiting participants for treatment research, potentially contributing to enhancing unbiased recruitment practices, reducing treatment gaps, and increasing engagement in digital intervention studies for improved mental health.

## Main text

### Methods

Since 2020, university students’ mental health has been surveyed within the Swedish arm of the World Health Organization World Mental Health International College Student (WHO-WMH-ICS) initiative [15, 16]. Participants are first-year university students enrolled in an undergraduate degree program at one of 15 participating universities in Sweden. From the fall of 2021, survey completers were invited to participate in a randomized controlled trial (RCT) offering transdiagnostic and tailored internet-based cognitive-behavioral therapy (ICBT) [17]. To gauge intervention research interest, survey participants were presented with a question at the end of the survey regarding their interest in participating in the treatment study. This article reports on expressed interest in participating in the treatment study, based on 9,140 individuals who responded to this question. Associations between expressed interest and later inclusion in the RCT will be reported elsewhere.

### Measures

The WHO-WMH-ICS survey consists of 11 sections, primarily derived from the Composite International Diagnosis Interview Screening Scales(CIDIC-SC) [18, 19], the Alcohol Use Disorder Identification Test (AUDIT) [20] and additional brief scales [15].

The variables analyzed included gender identity (male, female, non-binary) and age (18–20, 21–25, 26 and older) as well as 15 composite “treatment flag” variables indicating signs of mental disorder: attention deficit hyperactivity disorder (ADHD), major depressive disorder (MDD), panic disorder (PD), generalized anxiety disorder (GAD), any bipolar disorder (BPD), anger issues (ANG), post-traumatic stress disorder (PTSD), social anxiety disorder (SAD), binge eating disorder (BED), purging behavior (PB), alcohol use disorder (AUD), drug use disorder (DUD), suicidal ideation in the past 30 days (SI30), suicidal ideation in the past 12 months (SI12), and non-suicidal self-injury (NSSI). The treatment flag variables, generated by automatic algorithms within the survey based on mental health questions from several survey sections, indicated the *potential*, minimal fulfilment of criteria for a clinically defined diagnosis (e.g., MDD, PD, GAD) or behavior indicating a clinical condition (e.g., PTSD, SI30/SI12, NSSI). Thus, the presence of a positive treatment flag does not indicate the actual presence of a mental health diagnosis, but is rather an indicator of a potential minimal diagnostic fulfillment. Potential fulfilment of diagnostic criteria was indicated with a binary outcome, where 0 indicated the absence of the potential diagnosis and 1 indicated its presence.

The final survey question was not part of the original WHO-WMH-ICS survey, and was added by the researchers; the question assessed students’ willingness and interest to be contacted about participation in an intervention study. The item was worded as follows: “If we e-mail you about an additional study at the beginning of the next term, offering help and support for reduced mental health identified in this survey, would you consider participating?” The response alternatives were “Yes, I would consider participating”, “I might consider participating, not sure right now”, or “No, I would not consider participating”, coded as Yes, Maybe or No.

### Statistical analysis

Multinomial logistical regression was used to investigate associations between age, gender, treatment flags and interest in participating in intervention research. Data analysis was performed with Rstudio using the following packages: tidyverse [21], tidymodels [22], and nnet [23].

## Results

Participants’ ages ranged from 18 to 73 (*M* = 25.70, *Mdn* = 22.0, *SD* = 8.19), with 69% of participants identifying as female, 28% as male, and 3% as non-binary (see Table 1). The total number of positive treatment flags identified in the sample was 40,189 (*M* = 4.40, *Mdn* = 4, *SD* = 2.88). Participants were categorized into three age groups (18–20, 21–25, and 26 years and older) to achieve approximately equal group sizes. Due to rounding ages to the nearest whole year, the actual distribution deviated from the intended balance.

**Table 1:** Odds ratios^a^ for age, gender, and treatment flags about interest in future research participation among university students in Sweden 2020-23 (*N* = 9,140)

Sociodemographic characteristics	*n*	%	Yes OR [95% CI] *p*	Maybe OR [95% CI] *p*	No.
**Age (M = 25.7**,** SD = 8.19)**^**b**^	*n* = 9140	100%	1.00 [0.99–1.01] 0.64	0.98 [0.97–0.98] < 0.0001*	(ref)
18 to 20 years	*n* = 2330	25.5%	(ref)	(ref)	(ref)
21 to 25 years	*n* = 3465	37.9%	1.25 [1.04–1.50] < 0.05*	1.08 [0.90–1.30] 0.40	(ref)
26 years or older	*n* = 2960	32.4%	1.14 [0.95–1.37] 0.160	0·75 [0.63–0.91] < 0.05*	(ref)
**Gender** ^**b**^					
Male	*n* = 2552	27.9%	(ref)	(ref)	(ref)
Female	*n* = 6338	69.3%	1.49 [1.28–1.74] < 0.0001*	1.39 [1.19–1.62] < 0.0001*	(ref)
Non-Binary	*n* = 242	2.7%	1.86 [1.06–3.24] < 0.05*	1.55 [0.88–2.75] 0.13	(ref)
**Presence of treatment flag** ^c^					
ADHD	*n* = 3421	37.4%	1.15 [0.97–1.37] 0.117	1.03 [0.86–1.23] 0.74	(ref)
**Depression**	***n*** ** = 6400**	**70.0%**	1.62 [1.36–1.92] < 0.0001*	1.48 [1.24–1.76] < 0.0001*	(ref)
**Generalized anxiety**	***n*** ** = 3915**	**42.8%**	1.21 [1.01–1.45] < 0.05*	1.07 [0.89–1.29] 0.45	(ref)
**Panic disorder**	***n*** ** = 1238**	**13.5%**	1.46 [1.12–1.91] < 0.05*	1.56 [1.19–2.04] < 0.001*	(ref)
Bipolar disorder	*n* **=** 810	8.9%	1.13 [0.85–1.49] 0.40	0.95 [0.71–1.27] 0.74	(ref)
Anger	*n* **=** 1914	20.9%	1.04 [0.85–1.26] 0.72	1.00 [0.82–1.22] 0.98	(ref)
PTSD	*n* = 5231	57.2%	1.10 [0.94–1.28] 0.24	0.91 [0.78–1.06] 0.24	(ref)
**Social anxiety**	***n*** ** = 4241**	**46.4%**	1.48 [1.26–1.74] < 0.0001*	1.47 [1.25–1.74] < 0.0001*	(ref)
Binge	*n* = 2228	24.4%	1.04 [0.86–1.26] 0.68	1.08 [0.88–1.31] 0.46	(ref)
Purge	*n* = 1497	16.4%	1.18 [0.93–1.49] 0.17	0.89 [0.70–1.14] 0.36	(ref)
Alcohol use disorder	*n* = 2388	26.1%	1.06 [0.90–1.25] 0.49	1.06 [0.90–1.26] 0.46	(ref)
Substance use disorder	*n* = 553	6.1%	1.02 [0.73–1.42] 0.92	1.02 [0.72–1.44] 0.92	(ref)
30-day suicidal ideation	*n* = 2066	22.6%	0.98 [0.78–1.24] 0.89	0.92 [0.73–1.17] 0.51	(ref)
12-month suicidal ideation	*n* = 909	9.9%	1.36 [0.96–1.94] 0.08	1.18 [0.82–1.69] 0.38	(ref)
**Non-suicidal self-injury**	***n*** ** = 3378**	**37.0%**	1.42 [1.18–1.71] < 0.001*	1.31 [1.09–1.59] < 0.05*	(ref)

a. Residual deviance = 5410, AIC = 5530

b. Reference values are the youngest age group and male gender, respectively

c. Reference values for all treatment flags (binary variables) were defined as the absence of the treatment flag. The multinomial logistic regression model for treatment flags demonstrated a good fit to the data: *X*^2^ (*df* = 57, *N* = 9140) = 203.20, *p* < 0.001.

*Significance at *p* < 0.05

Those in the middle age group (21–25) were more likely, when compared to those in the youngest group, to respond ‘Yes’ (OR = 1.25, *p* < 0.05, 95% CI: 1.04–1.49), while those in the oldest group were significantly less likely to respond ‘Maybe’ (OR = 0.76, *p* < 0.05, 95% CI: 0.64–0.90), when compared to the youngest group. When age was assessed as a continuous variable, the multinomial logistic regression model showed that for each single year increase in age, participants were less likely to respond ‘Maybe’ (OR = 0.98, *p* < 0.001, 95% CI: 0.97–0.98) to the question regarding interest in intervention research participation. Compared to males, identifying as female was significantly associated with both ‘Yes’ (OR = 1.49, *p* < 0.0001, 95% CI: 1.28–1.74), and ‘Maybe’ (OR = 1.39, *p* < 0.0001, 95% CI: 1.19–1.62) responses. Identifying as non-binary was also significantly associated with responding ‘Yes’ (OR = 1.86, p < 0.05, 95% CI: 1.06–3.24), but not ‘Maybe’, when compared to male participants.

Five treatment flags were significantly associated with higher participation interest (Yes and/or Maybe). Qualifying for the MDD treatment flag was significantly associated with responding ‘Yes’ (OR = 1.62, *p* < 0.0001, 95% CI: 1.37–1.91) or ‘Maybe’ (OR = 1.47, *p* < 0.001, 95% CI: 1.24–1.75) regarding interest in participating in intervention research. Similarly, qualifying for the PD treatment flag was also significantly associated with both response categories: ‘Yes’ (OR = 1.52, *p* < 0.05, 95% CI: 1.17–1.98) or ‘Maybe’ (OR = 1.61, *p* < 0.001, 95% CI: 1.23–2.10). This also applied to the treatment flags for SAD: ‘Yes’ (OR = 1.46, *p* < 0.001, 95% CI: 1.24–1.71) or ‘Maybe’ (OR = 1.49, *p* < 0.001, 95% CI: 1.27–1.76), as well as the treatment flag for NSSI, ‘Yes’ (OR = 1.41, *p* < 0.001, 95% CI: 1.17–1.69) or ‘Maybe’ (OR = 1.31, *p* < 0.05, 95% CI: 1.09–1.57). In contrast, qualifying for the GAD treatment flag was significantly associated with a ‘Yes’ (OR = 1.21, p < 0.05, 95% CI: 1.01–1.44) response, but not the ‘Maybe’ (OR = 1.07, p = 0.45, 95% CI: 0.89–1.29) response category.

## Discussion

The investigation outcomes showed that age, gender identity and signs of mental disorder significantly contributed to a participant’s interest in engaging with mental health intervention research. Definite interest (“yes”) in digital intervention research participation was associated with older age, particularly being 21–25 years old, where possible interest (“maybe’) was less associated with being older than 26. This finding aligns with research on age effects in mental health research showing that middle-aged adults may be more willing than younger adults to participate; interestingly, adults over 60 appear less willing to engage with research compared to those between 18 and 29 years of age [10]. The contrast between middle-aged and older adults’ willingness may derive from older adults’ experiences of a variety of barriers to healthcare research participation compared to younger adults [24].

Regarding gender differences, it is known that participants identifying as female are more likely to participate in mental health research [12], in concordance with our findings. However, our investigation concerns expressed interest, not actual research engagement, where a discrepancy could exist among female participants. A recent meta-analysis on gender differences in research highlighted a systemic inadequate representation of females in actual research participation [25]. Future research should therefore investigate the potential gap between expressed interest and actual engagement in research among university students with diverse gender identities.

In terms of mental disorder symptomatology, previous research has identified depression, anxiety, and stress-based disorders as the most prevalent mental health concerns within the Swedish population [26, 27], whereas depression and general anxiety are the most prevalent disorders in the student population [15]. It is, therefore, not surprising that indications of these two disorders were associated with higher treatment interest. Interestingly, indications of social anxiety, panic disorder and self-harm were also highly associated with treatment study interest, despite varying prevalence rates. Notably, 10 of the treatment flags lacked significant associations with treatment study interest, despite the severity of these conditions and their associations with high levels of mental suffering; see, for example Strid et al. 2025 regarding eating disorders [28].

The strength of associations between mental health factors and treatment study interest may be affected by the population prevalence of a disorder, variations in stigma levels [29], and barriers identified earlier such as the preferences for self-help, for seeking support from friends or family and feelings of embarrassment in seeking help [7]. Although digital interventions are known to attract individuals with high levels of stigma and reluctance to engage with ordinary in-person treatment [2, 3] the question on treatment interest to our participants did not indicate that the treatment study in question would be in digital format. Future research should consider the influence of mental disorder symptoms and diagnosis on actual intervention research participation and, potentially, also explore possible differential effects of digital versus in-person treatment study offers.

## Conclusion

We found that signs of several common mental health conditions (MDD, PD, SAD, NSSI, GAD), along with older age and non-male gender identity, predicted students’ expressed interest in intervention research. Understanding these patterns can guide more inclusive recruitment strategies to reduce bias and narrow treatment gaps. Future research should investigate various factors that affect participation and continue to explore how expressed interest translates into actual participation and treatment engagement.

Strengths and limitations.

The major strength of our study was its large sample and the broad scope of variables analyzed in relation to intervention study interest. One limitation concerns the validity of treatment flags in relation to diagnoses, as a psychometric investigation into this relationship has not yet been undertaken. Diagnoses based on diagnostic DSM-5 algorithms from data collected within the WHO-WMH-ICS initiative have recently been reported for the first time [15], and future research will need to validate the calculation of the treatment flag algorithms. A second limitation concerns our focus on expressed interest in treatment, without considering actual treatment participation, as well as treatment retention over time. This research topic will be explored in future analyses which will be initiated once initial RCT outcome analyses have been conducted [17]. A third limitation is that our findings are restricted to a sample from one country. Further investigation in additional population samples is warranted.

## Data Availability

Unidentified data are available on reasonable request to the Principal Investigator (PI), author AHB.
